# Reversible SAHH inhibitor protects against glomerulonephritis in lupus-prone mice by downregulating renal α-actinin-4 expression and stabilizing integrin-cytoskeleton linkage

**DOI:** 10.1186/s13075-019-1820-3

**Published:** 2019-01-29

**Authors:** Shijun He, Xing Liu, Zemin Lin, Yuting Liu, Lei Gu, Hu Zhou, Wei Tang, Jianping Zuo

**Affiliations:** 1 Laboratory of Immunopharmacology, State Key Laboratory of Drug Research, Shanghai Institute of Materia Medica, Chinese Academy of Sciences, Shanghai, 201203 China; 2 University of Chinese Academy of Sciences, No.19A Yuquan Road, Beijing, 100049 China; 3 Department of Analytical Chemistry and CAS Key Laboratory of Receptor Research, Shanghai Institute of Materia Medica, Chinese Academy of Sciences, Shanghai, 201203 China

**Keywords:** SAHH inhibitor, Lupus nephritis, α-Actinin-4, Integrin-linked kinase, Integrin, Focal adhesion

## Abstract

**Background:**

Glomerulonephritis is one of the major complications and causes of death in systemic lupus erythematosus (SLE) and is characterized by glomerulosclerosis, interstitial fibrosis, and tubular atrophy, along with severe persistent proteinuria. DZ2002 is a reversible S-adenosyl-l-homocysteine hydrolase (SAHH) inhibitor with potent therapeutic activity against lupus nephritis in mice. However, the molecular events underlying the renal protective effects of DZ2002 remained unclear. This study is designed to uncover the molecular mechanisms of DZ2002 on glomerulonephritis of lupus-prone mice.

**Methods:**

We conducted a twice-daily treatment of DZ2002 on the lupus-prone NZB/WF1 mice, and the progression of lupus nephritis and alteration of renal function were monitored. The LC-MS-based label-free quantitative (LFQ) proteomic approach was applied to analyze the kidney tissue samples from the normal C57BL/6 mice and the NZB/WF1 mice treated with DZ2002 or vehicle. KEGG pathway enrichment and direct protein-protein interaction (PPI) network analyses were used to map the pathways in which the significantly changed proteins (SCPs) are involved. The selected proteins from proteomic analysis were validated by Western blot analysis and immunohistochemistry in the kidney tissues.

**Results:**

The twice-daily regimen of DZ2002 administration significantly ameliorated the lupus nephritis and improved the renal function in NZB/WF1 mice. A total of 3275 proteins were quantified, of which 253 proteins were significantly changed across normal C57BL/6 mice and the NZB/WF1 mice treated with DZ2002 or vehicle. Pathway analysis revealed that 13 SCPs were involved in tight junction and focal adhesion process. Further protein expression validation demonstrated that DZ2002-treated NZB/WF1 mice exhibited downregulation of α-actinin-4 and integrin-linked kinase (ILK), as well as the restoration of β1-integrin activation in the kidney tissues compared with the vehicle-treated ones.

**Conclusions:**

Our study demonstrated the first evidence for the molecular mechanism of SAHH inhibitor on glomerulonephritis in SLE via the modulation of α-actinin-4 expression and focal adhesion-associated signaling proteins in the kidney.

**Electronic supplementary material:**

The online version of this article (10.1186/s13075-019-1820-3) contains supplementary material, which is available to authorized users.

## Background

Systemic lupus erythematosus (SLE) is an autoimmune disorder characterized by widespread loss of immune tolerance to self-antigen affecting multiple organs of which lupus nephritis (LN) is the most common and a predictor of poor overall outcome of SLE patients [1, 2]. Proteinuria is the clinical manifestation of a defective glomerular filtration and an important causative factor for the progression of renal failure in patients with LN [3].

Glomerular filtration barrier consists of fenestrated endothelium, glomerular basement membrane (GBM), and terminally differentiated podocytes. Podocytes are susceptible to injury as seen in many forms of glomerular diseases, including LN, focal segmental gomerulosclerosis (FSGS), and membranous glomerulopathy [4, 5]. Podocyte morphological changes, including foot processes (FPs) effacement and detachment from GBM, are key events in progressive glomerular failure.

α-actinin is an actin-bundling protein thought to have essential roles in helping to form the anchoring complex for the ends of actin stress fibers [6, 7]. α-actinin-4 (encoded by *ACTN4* gene) is highly enriched in podocyte FPs, and dysregulation of this protein is one of the primary events that occur in the early stage of several nephrotic syndromes [6, 8, 9]. Accumulated evidence demonstrated that overexpression of α-actinin-4 in mice leads to rearrangement of the actin cytoskeleton and subsequent FPs effacement associated with renal insufficiency and proteinuria [10, 11].

α3β1 is the predominant integrin expressed along the basal surface of podocytes and functions as receptors transducing signals on contact with the extracellular matrix (ECM) [12]. The cytoplasmic domain of β1 is the most common isoform and interacts with proteins of the focal adhesion complex including α-actinin, talin, and numerous signaling proteins, including integrin-linked kinase (ILK), focal adhesion kinase (FAK), and adaptor proteins [13]. Particularly, ILK has been shown to play an essential role in the establishment and maintenance of integrin-actin connection [14], and stable overexpression of ILK in murine podocytes caused reduced matrix adhesion and led to considerable phenotype alteration in murine progressive glomerulosclerosis [15].

SAHH and its substrate S-adenosyl-l-homocysteine (SAH) are deeply involved in the process of transmethylation mediated by S-adenosylmethionine (SAM) [16, 17], and the immunosuppressive properties of SAHH inhibitors have been well documented. DZ2002 [methyl-(adenin-9-yl)-2-hydroxybutanoate] is a reversible type III SAHH inhibitor, and it has been shown to exert therapeutic effects on lupus-prone mice, by regulating Toll-like receptor (TLR)-triggered antigen-presenting cells (APCs) functions [18]. Moreover, in our latest study, we reported that topical administration of DZ2002 attenuated psoriasis partly by interfering the abnormal activation and differentiation of keratinocytes in skin lesions [19].

Advances in mass spectrometry (MS) enable the identification and quantification of thousands of proteins in complex biological samples, in a single run during the last two decades. Pharmacoproteomics is the use of proteomic technologies in drug discovery and development [20]. Along with pharmacogenomics and pharmacogenetics, pharmacoproteomics plays an important role in autoimmune diseases related drug targets’ identification and validation [21]. Recently, there are many studies using pharmacoproteomics to search for molecular changes in sorts of biological specimens from patients with SLE [22–27].

In the present study, we performed a reproducible, well-controlled, label-free quantitative (LFQ) proteomic analysis of kidney tissue samples from normal and lupus-prone mice treated with DZ2002 or vehicle. The results of the LFQ proteomics and the subsequent validation experiments provided novel evidence that the molecular changes of focal adhesion and cytoskeleton of podocytes may be associated with the therapeutic mechanisms of DZ2002 on glomerulonephritis (Additional file 1). Furthermore, the current study also highlighted a potential mechanism for the tissue-protective effects SAHH inhibitors on autoimmune diseases.

## Methods

### Animals

Female NZB/WF1 mice purchased from the Jackson Laboratory and female C57BL/6 mice purchased from Shanghai Laboratory Animal Center of the Chinese Academy of Sciences were used for this investigation. All mice were housed in a pathogen-free facility and were housed in clean-grade animal cabin with free access to standard laboratory water and food, and kept in a 12-h light/dark cycle with controlled humidity (60–80%) and temperature (22 ± 1 °C).

The animal experiment was carried out in strict accordance with the institutional ethical guidelines on animal care and was approved by the Institute Animal Care and Use Committee (IACUC) at the Shanghai Institute of Materia Medica, Chinese Academy of Sciences (IACUC protocol # 2012-06-ZJP-17 for NZB/WF1 mice, IACUC protocol # 2012-12-ZJP-18 for C57BL/6 mice).

### Experimental design

NZB/WF1 mice, an established model of LN, were chosen as the glomerulonephritis model for this study.

Female NZB/WF1 mice (*n* = 27) at 24 weeks old were randomly divided according to proteinuria level into 3 groups (*n* = 9 per group): vehicle (ddH_2_O), prednisolone (PNS, 2 mg/kg, once daily), and DZ2002 (8 mg/kg, twice daily, total dosage). Mice were received oral intragastric administration for 15 weeks. During the treatment, individuals were euthanized, followed by serum and kidney collection if their urine protein concentration was > 10 mg/mL for 2 consecutive weeks, accompanied by weight loss > 20%. In this case, the last known values for urinary protein were carried forward, and the kidney and serum were collected for further assessments [28].

Age-matched female C57BL/6 mice were used as a normal control in the quantitative analysis of label-free proteomics of kidney samples, as well as in the following renal immunohistochemical evaluation and semi-quantification of proteins.

To characterize the alteration of pivotal glomerular cytoskeleton proteins and focal adhesion components during the progression of glomerulonephritis in lupus-prone mice, female NZB/WF1 mice of different ages (16 weeks, 24 weeks, and 42 weeks included) were used. All of the mice were euthanized at the same time, and the kidneys were isolated for further assessment.

### Sample collection and analysis

Urine from individual mice was collected weekly by the method described previously [29, 30]. Blood samples were obtained as the mice were sacrificed.

At the end of the 15-week treatment, the kidneys were dissected and the left ones were immersion-fixed in 10% neutral-buffered formalin, and embedded in paraffin, for histopathological evaluation and immunohistochemical examination of protein distribution. The right kidneys were snap-frozen and stored at − 80 °C for Western blot analyses and subsequent proteomic analysis.

### Evaluation of renal function

Concentration of urinary protein was determined by a Coomassie Brilliant Blue dye-binding assay.

Blood urea nitrogen (BUN) and creatinine concentrations were determined using a HITACHI-7080 automatic biochemical analyzer (Hitachi High-Technologies Corporation, Tokyo, Japan).

### Histopathological assessment

Sections (3 μm) were stained with hematoxylin and eosin (H&E) and periodic acid-Schiff (PAS) for pathological evaluation. Pathological scores of each mouse were calculated according to the glomerular, interstitial, vascular lesions, and matrix expansion according to reported criteria [18].

H&E stained sections were captured using ImageScope (Aperio Technologies, Inc. Vista, CA, USA) at × 200 magnification, and PAS-stained sections were acquired using Leica DM RXA2 (Leica Microsystems AG, Wetzlar, Germany) at × 400 magnification.

### Glomerular and mesangial area calculation

The kidney sections were stained with PAS for assessment of the total glomerular tuft area and glomerular basement membrane (mesangial) area. The mean glomerular tuft area was determined by counting all available glomeruli within each kidney section. The mesangial matrix area was calculated as the PAS-positive area compared to the total glomerular tuft area using all available glomeruli within each kidney.

### Immunohistochemistry

The sections of the kidneys were stained using anti-mouse α-actinin-4 (Cell Signaling Technology, Beverly, MA, USA) and anti-mouse Wilms’ tumor 1(WT-1, Abcam, Cambridge, UK). Hematoxylin was used as counter-staining. The staining images were acquired using an optical microscope (Leica DM RXA2, Leica Microsystems AG, Wetzlar, Germany) at × 400 magnification. For quantitative determination of podocyte numbers, the WT-1-positive cells were counted in 10 randomly chosen glomeruli under high power (× 400 magnification) in each mouse.

### Protein extraction, digestion, and peptide purification for proteomic study

The kidney tissue samples for proteomic analysis were rinsed several times to remove the blood using pre-cold phosphate-buffered saline (PBS), and the proteins were extracted from the kidney tissue with SDT lysis buffer (100-mM dithiothreitol [DTT], 4% sodium SDS, 100-mM Tris-HCl, pH 7.6) using mechanical homogenization followed by ultrasonication. The protein concentration was determined using a tryptophan-fluorescence assay [31] and confirmed by 12% sodium dodecyl sulfate-polyacrylamide gel electrophoresis (SDS-PAGE). An equal amount of protein (50 μg) from each sample was digested using the filter-aided sample preparation (FASP) method [32]. Briefly, an equal amount of protein (50 μg) from each sample was mixed with UA solution (8 M urea in 100 mM Tris-HCl, pH 8.5) in a 10-kDa filter (Microcon, Millipore, Germany) and centrifuged at 12,000 g for 25 min at 20 °C. Proteins were alkylated with iodoacetamide (final concentration 50 mM IAA) at room temperature for 30 min. Proteins were digested by trypsin (Promega Corporation, Madison, WI, USA) at an enzyme to substrate ratio of 1:50 (*w*/*w*) at 37 °C for 16 h, and 200 μL of 50 mM NH_4_HCO_3_ (Sigma-Aldrich Corporation, St. Louis, MO, USA) was added to elute the resulting peptides. Digested peptides were desalted and evaporated to dryness in a Speed-Vac sample concentrator. Finally, the peptides were subjected to LC-MS/MS analysis.

### LC-MS/MS analysis

LC-MS/MS was performed as previously described with some modifications [33, 34]. LC-MS/MS analysis was carried out by coupling an Easy nano-UPLC1000 (Thermo Fisher Scientific, Bremen, Germany) to a Q Exactive (Thermo Fisher Scientific, Bremen, Germany). Peptides (2 μg) were loaded on to an in-house packed analytical column (75 μm i.d. × 15 cm, ReproSil-Pur C18-Pur, 3 μm, Dr. Maisch GmbH, Ammerbuch, Germany), operating a 240-min gradient at a flow rate of 300 nL/min. Mobile phase A consisted of 0.1% formic acid, and mobile phase B consisted of 0.1% formic acid in acetonitrile. The ionization of the resulting peptides was conducted at a capillary temperature of 320 °C using 2.2 kV ion spray voltage. The mass spectrometer operated in data-dependent acquisition mode. Full scan MS1 spectra (300–1500 *m*/*z*) were acquired in the Orbitrap for a maximum ion injection time of 60 ms at a resolution of 70,000 (at *m*/*z* = 200), and the subsequent MS/MS analyses were performed in the same analyzer at a resolution of 17,500 (at *m*/*z* = 200). Up to 20 precursor ions could be selected for fragmentation, whereby the isolation window was set to 2 *m*/*z* and normalized collision energy (NCE) to 28%.

### Database searching and analysis

For protein identification and quantification, we used MaxQuant [35] (http://maxquant.org/, version 1.5.1.0) to analyze the MS data. Carbamidomethyl cysteine (C) was set as a fixed modification, and oxidation (*M*, + 15.99492 Da) was set as a variable modification. Proteins were identified by searching MS and MS/MS data of peptides against a decoy mouse proteome sequence (*Mus musculus* C57BL/6; UP000000589; Uniprot proteome database). Trypsin/P was selected as the digestive enzyme with two potential missed cleavages. The false discovery rate (FDR) for peptides and protein groups was rigorously controlled to be < 1%. FDR was calculated by the number of hits from the reverse database divided by the number of forward hits [36]. Label-free quantification was carried out in MaxQuant using intensity determination and a normalization algorithm. The “LFQ intensity” of each protein in different samples was calculated as the best estimate, satisfying all of the pairwise peptide comparisons, and this LFQ intensity was almost on the same scale of the summed-up peptide intensities [37]. Perseus software (version 1.4.1.3) was used to further process the protein LFQ intensity lists. The *p* value of LFQ intensity of each protein between any two groups by two-tailed Student’s *t* test was also calculated by the Perseus program, and *p* value < 0.05 was set as a cutoff criterion for a significant change.

The mass spectrometry proteomics data have been deposited to the ProteomeXchange Consortium via the PRIDE [38] partner repository with the dataset identifier PXD010059.

### Western blotting

Ten-milligram tissue of each kidney was homogenized and directly lysed using sodium dodecyl sulfate (SDS) sample buffer containing protease inhibitor cocktail (Roche Life Science, Mannheim, Germany), and the protein concentrations were measured using a BCA protein assay kit (Pierce, Rockford, IL, USA).

Equal amounts of protein were separated on 10% or 12% SDS-PAGE and Western blotted with antibodies to mouse α-actinin 4 (Cell Signaling Technology, Beverly, MA), integrin-linked kinase, β1-integrin, phosphorylated β1-integrin (T788 + T789), Wilms’ tumor 1, and α-tubulin (Abcam, Cambridge, UK). The densities of the bands were quantified with a computerized densitometer (ImageJ 1.42, National Institutes of Health, Bethesda, MD).

### Statistical analysis

One-way ANOVA followed by Dunnett’s multiple comparison test was used to compare parameters involving multiple groups, with GraphPad Prism 7.0 (GraphPad Software, San Diego, CA, USA) statistical software. *P* values less than 0.05 were considered significant.

## Results

### DZ2002 treatment reduced proteinuria and preserved renal function in NZB/WF1 mice

**Fig. 1:**
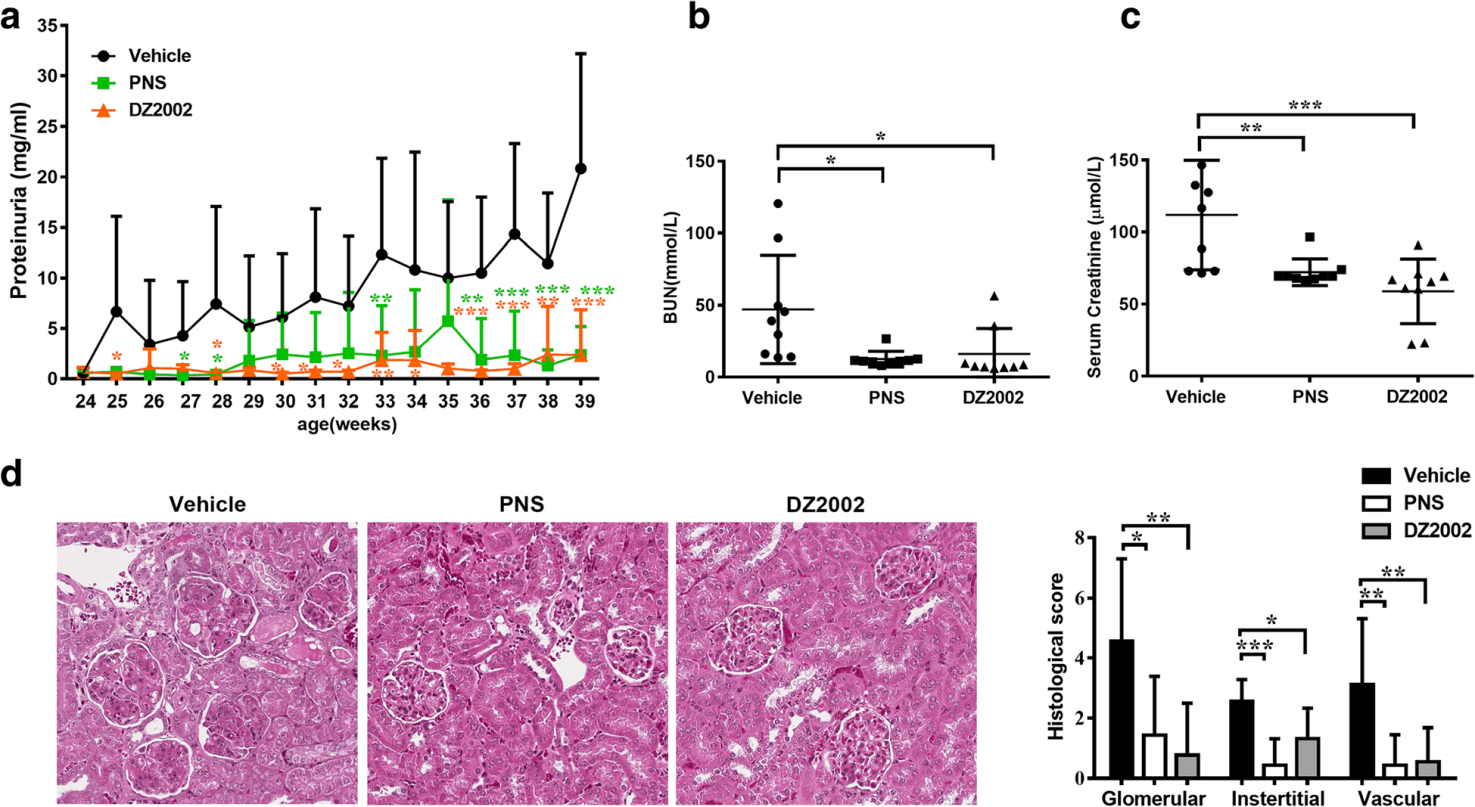
DZ2002 treatment reduced proteinuria and preserved renal function in NZB/WF1 mice. Twenty-four-week-old female NZB/WF1 mice were orally treated with vehicle (H_2_O), DZ2002 (8 mg/kg, twice a day), and PNS (2 mg/kg) for 15 weeks (*n* = 9 per group). **a** Levels of proteinuria. Serum levels of blood urea nitrogen (BUN) (**b**) and creatinine (**c**) measured at the end of the experiment. Each dot represented data for individual mouse. **d** Left: Representative kidney sections stained with H&E (× 200 magnification). Right: Glomerular, interstitial, and vascular pathologic changes were scored from (0) to (4+) as described in the “Methods” section. Statistics results derived from individual mouse of each group (*n* = 5 per group). Data were shown as the mean ± SD. *P < 0.05, **P < 0.01, and ***P < 0.001 versus vehicle

Proteinuria was measured in NZB × NZW F1 mice to assess nephritis-associated loss of renal function. We optimized the previous dose regimen [18] of DZ2002 to a twice-daily administration, with view to the reversible inhibitory property and a relative shorter biological half-life of DZ2002. As expected, both DZ2002 and PNS treatment retarded the progression of proteinuria in contrast to vehicle-treated mice (Fig. 1a). Additionally, DZ2002 treatment prevented the worsening of renal function, represented by a significant decrease in BUN and serum creatinine levels compared with the vehicle group (Fig. 1b and c). Following 15 weeks of DZ2002 treatment, mice demonstrated reductions in diffuse proliferation of glomerular cells, proteinaceous deposits in the mesangium, and tubular cast formation.

### Quantitative analysis of label-free proteomic data

**Fig. 2:**
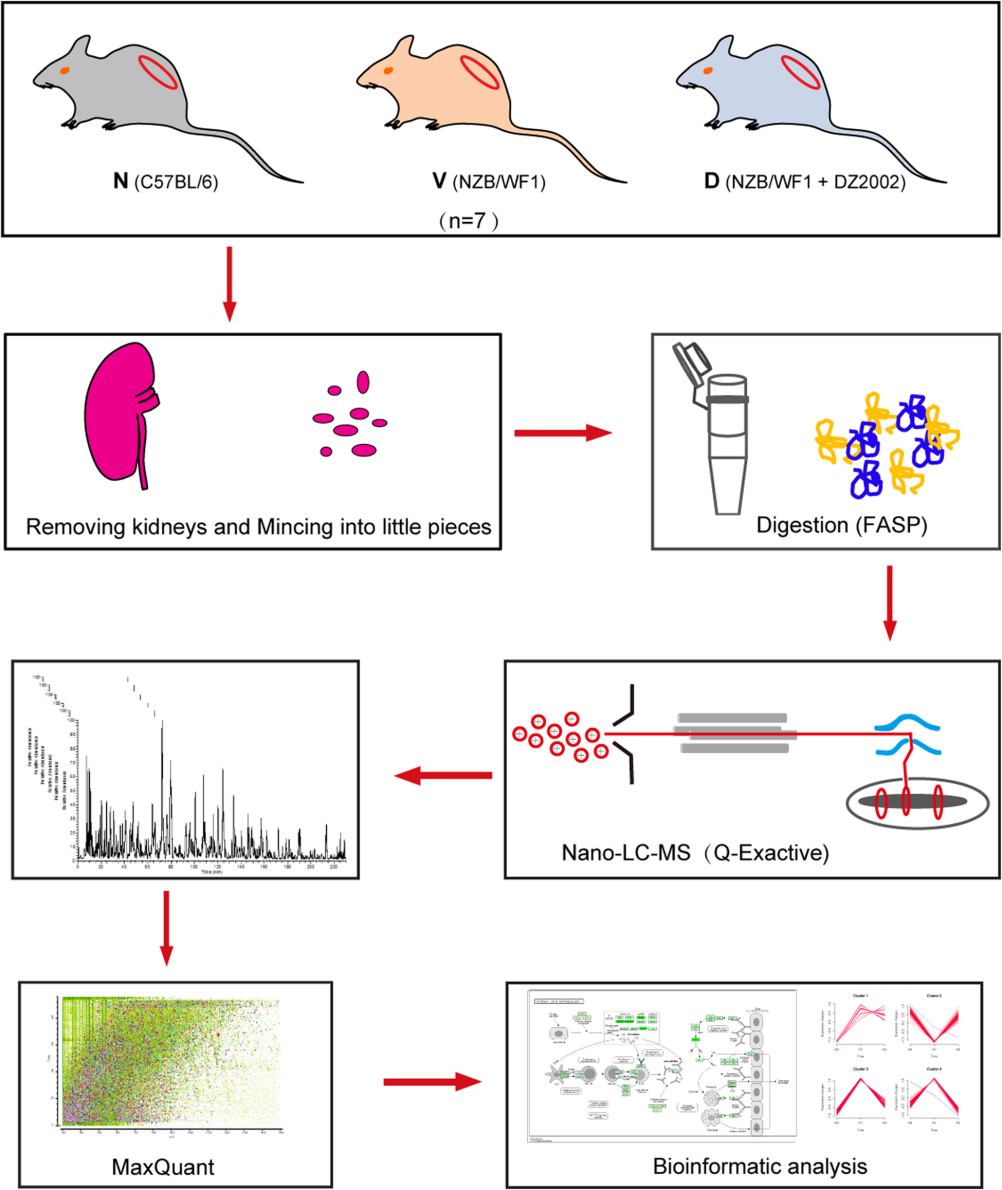
Flow chart of the label-free quantitative proteomic study. Flow chart of the proteomic analysis of the kidney tissues from mice of three groups. Proteins were extracted and digested by the FASP method. The resulting peptides were analyzed by high-resolution nano-LC-MS/MS. Peptides and proteins were quantified with the label-free algorithm in MaxQuant software. The differentially proteins were further analyzed by pathway mapping and protein-protein interaction network analyses. N, C57BL/6 mice; V, vehicle mice in the treatment experiment; D, DZ2002-treated mice in the treatment experiment

To explore the molecular mechanism of DZ2002 on lupus-prone mice, the kidneys of three groups were collected (*n* = 7 per group): normal group (*N*, age-matched female C57BL/6 mice), vehicle-treated group (*V*, vehicle-treated NZB/WF1 mice in the therapeutic experiment), and DZ2002-treated group (*D*, DZ2002-treated NZB/WF1 mice in the therapeutic experiment). The tissue protein extracts were analyzed using label-free quantitative proteomic workflow (Fig. 2). The filter-aided sample preparation (FASP) was used to process all of the samples. Tryptic peptides were analyzed by LC-MS/MS on a quadrupole Exactive mass spectrometer. The relative LFQ intensity of each protein across the individual samples was obtained using MaxQuant [35]. The consistency and reproducibility of this proteomic platform has already been demonstrated in our previous studies [33].

**Fig. 3:**
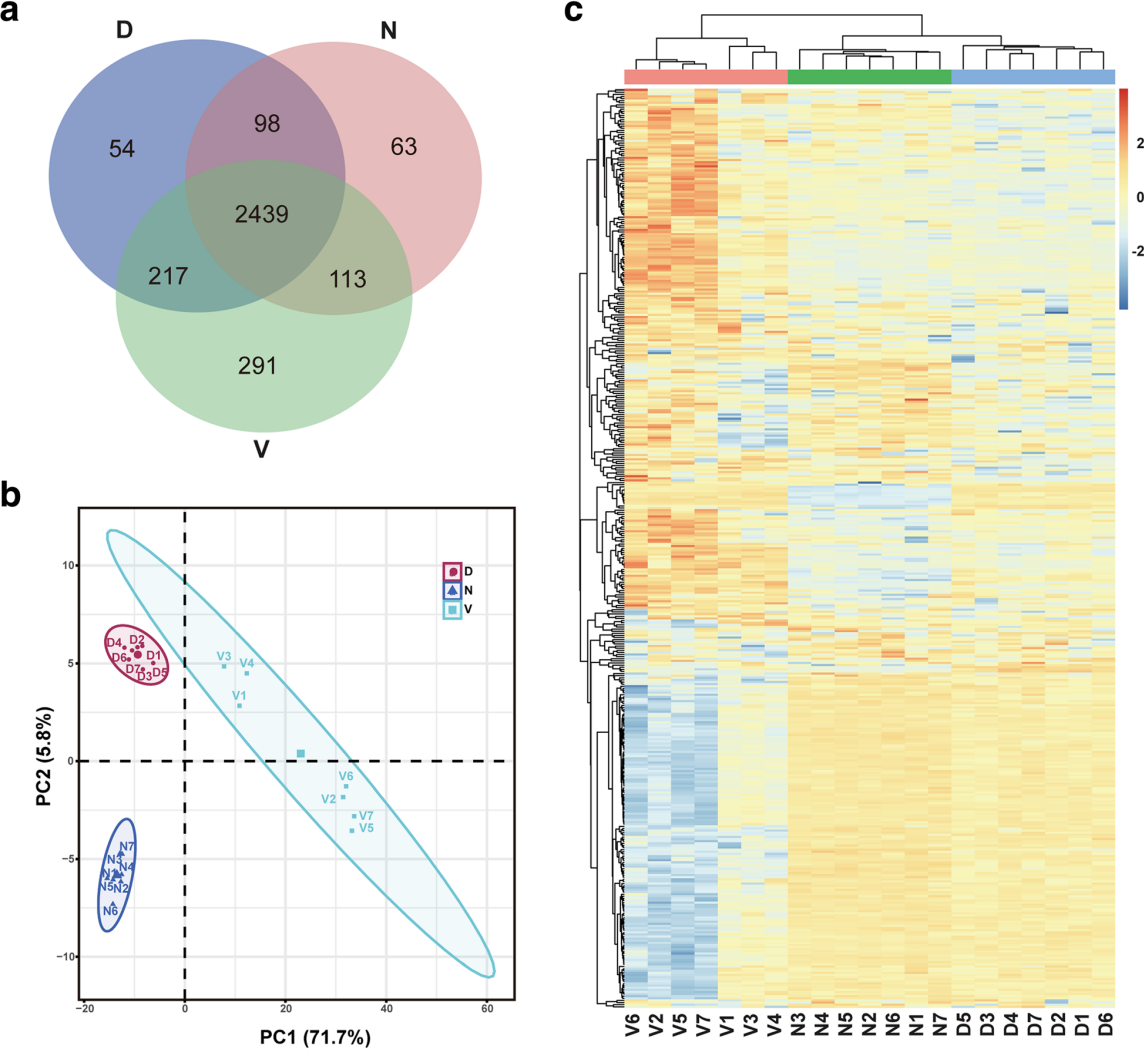
Quantitative proteomic analysis. **a** Venn diagram shows the shared and uniquely identified proteins in three groups. **b** The principal component analysis of three groups. **c** The hierarchical clustering analysis of three groups. N, C57BL/6 mice; V, vehicle mice in the treatment experiment; and D, DZ2002-treated mice in the treatment experiment

To provide highly confident protein identification, the analysis of all raw files was based on stringent criteria of < 1% FDR at both peptide and protein group levels. As shown in Additional file 2: Table S1, the mean and SD value of MS scans, MS/MS scans, identified MS/MS scans, percentage of identified MS/MS scans, and unique peptide sequences, from the entire 21 LC-MS/MS runs, are 18,757 ± 163, 98,057 ± 492, 26,764 ± 522, 20.70 ± 0.39%, and 13,476 ± 226, respectively. The relative standard deviation value of each record is less than 5%, which suggests the system variation of each LC-MS/MS run was well controlled. These results indicate that these high-quality data sets are suitable for comparative label-free analysis. In our analysis, there were 27,458 unique peptides identified, corresponding to 2713, 3060, and 2808 protein in the normal, vehicle, and DZ2002-treated kidney samples as shown in Fig. 3a. Taken together, 3275 protein groups were detected from all of the three groups.

To compare the LFQ intensity of each protein across all 21 individual samples, a violin plot was generated in Additional file 1: Figure S1a. The mean of log_2_ LFQ intensity (about 23.41 ± 0.01 of each sample) is almost at the same level across the samples, indicating there were no biases toward different samples in LFQ analysis. The correlation co-efficient of the LFQ intensities between two LC-MS/MS runs was higher than 0.80 (Additional file 1: Figure S1b). Relative label-free quantitation profiling was highly reproducible between biological replicates inside each cohort or between the LC-MS/MS runs from different cohorts.

Furthermore, Student’s *t* test analysis was performed to identify differentially expressed proteins between any two groups. Proteins with *p* value of Student’s *t* test (vehicle-treated versus normal group and DZ2002-treated versus vehicle-treated group) lower than 0.01 and fold change of the two compares higher than 1.4 or lower than 0.7 were considered as significant changed proteins (SCPs). In view of the therapeutic effects of DZ2002, the SCPs should also meet the criteria of *p* value of Student’s *t* test (DZ2002-treated group versus normal group) higher than 0.05. In total, 253 SCPs were obtained under the above strict filtration criteria (Additional file 2: Table S2). Principal component analysis (PCA) was used to analyze the SCPs. PCA score plot displayed vehicle-treated group were clearly separated from normal group and DZ2002-treated group by the first principal components, and this explained 71.7% of the variability of the identified proteins (Fig. 3b). A heat-map was also shown in Fig. 3c from hierarchical clustering analysis (HCA) of the SCPs intensities. As demonstrated in the diagrams of the HCA, the normal group, vehicle-treated group, and DZ2002-treated group were classified into three respective clusters, predicting the profound proteomic changes in the vehicle-treated and DZ2002-treated groups.

### Functional analysis of identified SCPs

The SCPs were performed to map KEGG pathway, and the enrichment of these proteins in each pathway was obtained by calculating the probability using hypergeometric distribution. As shown in Additional file 1: Figure S2a, 14 pathways were enriched with *p* value *<* 0.05 and enriched protein numbers were more than ten, including tight junction, focal adhesion, phagosome, carbon metabolism, lysosome, complement and coagulation cascades, cardiac muscle contraction, retrograde endocannabinoid signaling, Huntington’s disease, thermogenesis, non-alcoholic fatty liver disease (NAFLD), Alzheimer’s disease, Parkinson’s disease, and oxidative phosphorylation.

Furthermore, gene ontology (GO) was used to annotate the cellular compartments and molecular functions of the SCPs. Proteins were linked to at least one annotation term each within the GO cellular component, biological process, and molecular categories, and a single protein is usually involved in multiple molecular and cellular functions in GO. The major cellular component categories were respiratory chain (enriched *p* value 7.51E−46, enriched proteins 34), mitochondrial respiratory chain (enriched *p* value 5.31E−46, enriched proteins 33) (Additional file 1: Figure S2b). The major biological process categories were electron transport chain (enriched *p* value 5.36E−24, enriched proteins 21), mitochondrial respiratory chain complex I assembly (enriched *p* value 1.39E−25, enriched proteins 18), mitochondrial respiratory chain complex I biogenesis (enriched *p* value 1.39E−25, enriched proteins 18) (Additional file 1: Figure S2c). The most common molecular functions were hydrogen ion transmembrane transporter activity (enriched *p* value 1.87E−15, enriched proteins 16), cofactor binding (enriched *p* value 1.52E−7, enriched proteins 15), and NADH dehydrogenase activity (enriched *p* value 5.88E−23, enriched proteins 15) (Additional file 1: Figure S2d).

**Fig. 4:**
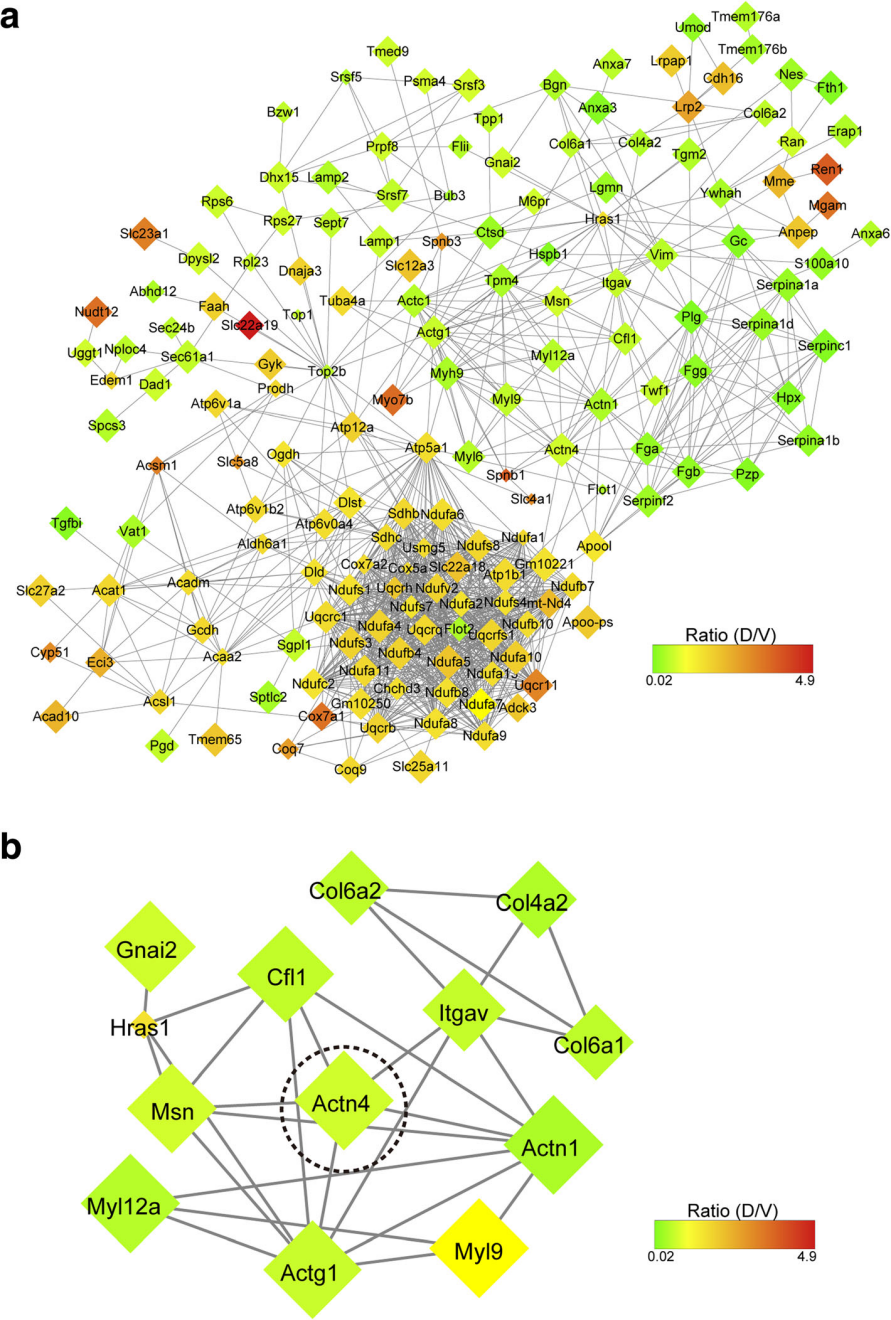
Functional analysis of significantly changed proteins (SCPs). **a** PPI network analysis of all the 253 SCPs. **b** PPI network analysis of SCPs involved in both tight junction pathway and focal adhesion pathway. Actn4 is pointed out by a dashed circle. The gene shape size represents the *p* value of comparison between model group and drug group by two-tailed Student’s *t* test in the negative logarithmic scale, ranging from the lowest significance (small) to the strongest (big). The color of each gene represents the ratio of D/V, ranging from the minimum value (green) to the maximum value (red). Disconnected genes are hidden

To dissect the possible roles of these SCPs in the therapeutic mechanism of DZ2002, we used the STRING protein-protein interaction database (http://string-db.org) to map all of these types of changed proteins into their direct protein-protein interaction (PPI). The resulting network was visualized by Cytoscape 3.1.1 and included 214 nodes and 1024 edges (Fig. 4a). We further combined the KEGG pathway enrichment results with the PPI network. There were 13 SCPs involved in tight junction and focal adhesion pathways. Protein-protein interactions of these proteins were showed in Fig. 4b, among which α-actinin-4 may contribute to the protective effect of DZ2002 against glomerulonephritis in lupus-prone mice, and was selected for further biological validations.

### DZ2002 treatment attenuated glomerulosclerosis in lupus-prone mice

Glomerulosclerosis is a common histologic pattern of kidney injury, often associated with progressive chronic kidney disease. Abnormal expression of α-actinin-4 is thought to be implicated in the pathogenesis of FSGS, characterized by sclerosis occurring in a portion of the glomerulus.

**Fig. 5:**
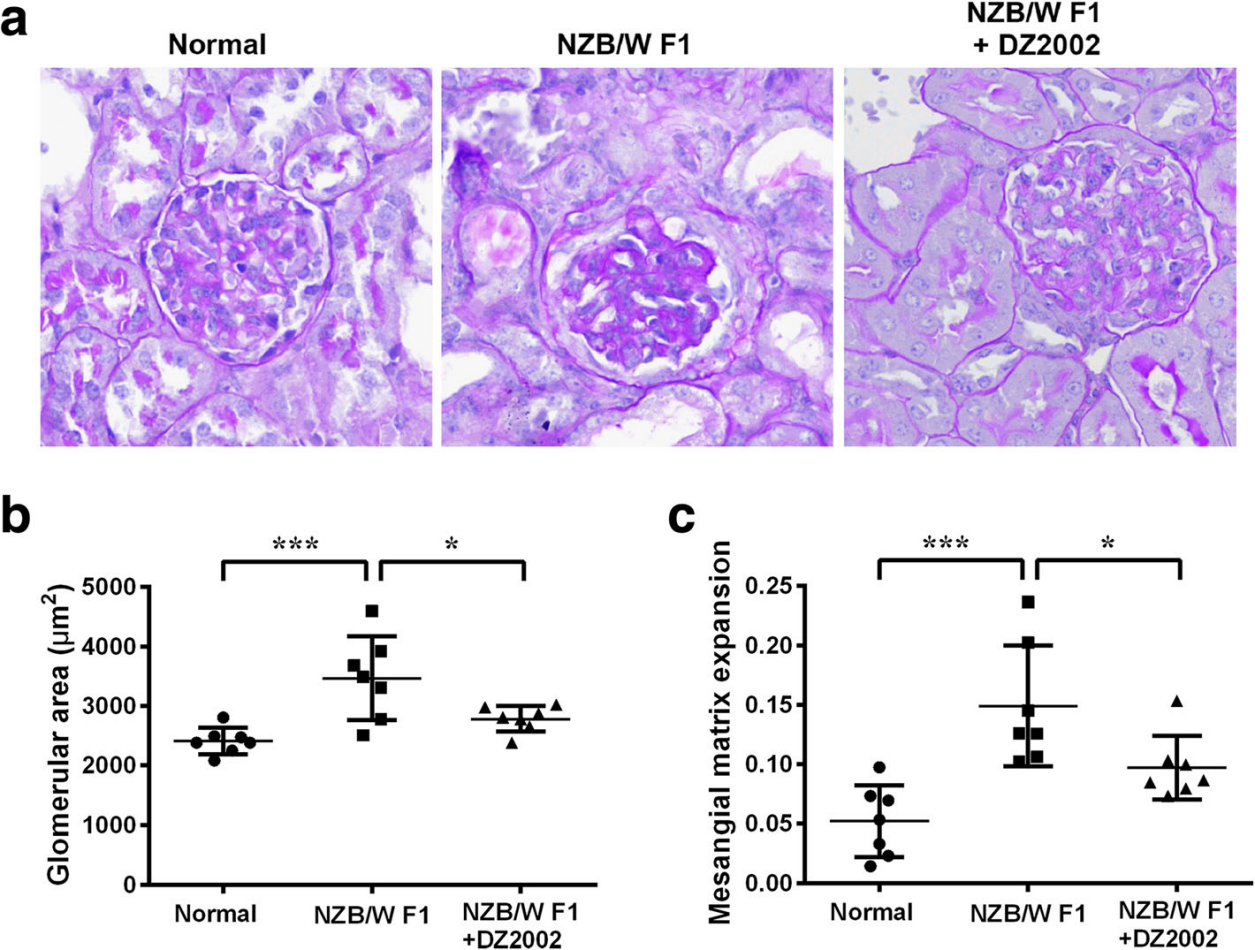
DZ2002 treatment attenuated glomerulosclerosis in lupus-prone mice. **a** Representative PAS-stained kidney section (× 400 magnification). **b** The glomerular tuft area was averaged from all available glomeruli per kidney section to give *n* = 1, using one kidney section per mouse. **c** Mesangial matrix fraction was calculated as the ratio of PAS-positive area to total glomerular tuft area and averaged from all available glomeruli per kidney section to give *n* = 1. Normal, age-matched female C57BL/6 mice; NZB/WF1, vehicle mice in the treatment experiment; and NZB/WF1+DZ2002, DZ2002-treated mice in the treatment experiment. Each dot represented data for individual mouse (*n* = 7 per group). Data were shown as the mean ± SD. *P < 0.05, ***P < 0.001 versus NZB/WF1 group

Further examination of PAS-stained kidney sections indicated that the glomerular area in NZB/WF1 mice increased by 43.9% compared to normal mice, and DZ2002 treatment significantly normalized the increase to 15.5% (Fig. 5a and b). Quantification result indicated an expansion of mesangial matrix in NZB/WF1 mice by 2.86-fold compare with normal mice, and the extent of expansion was restrained to 1.86-fold by DZ2002 treatment (Fig. 5c).

### DZ2002 treatment reduced aberrant distribution and accumulation of α-actinin-4 in the glomeruli of NZB/WF1 mice

**Fig. 6:**
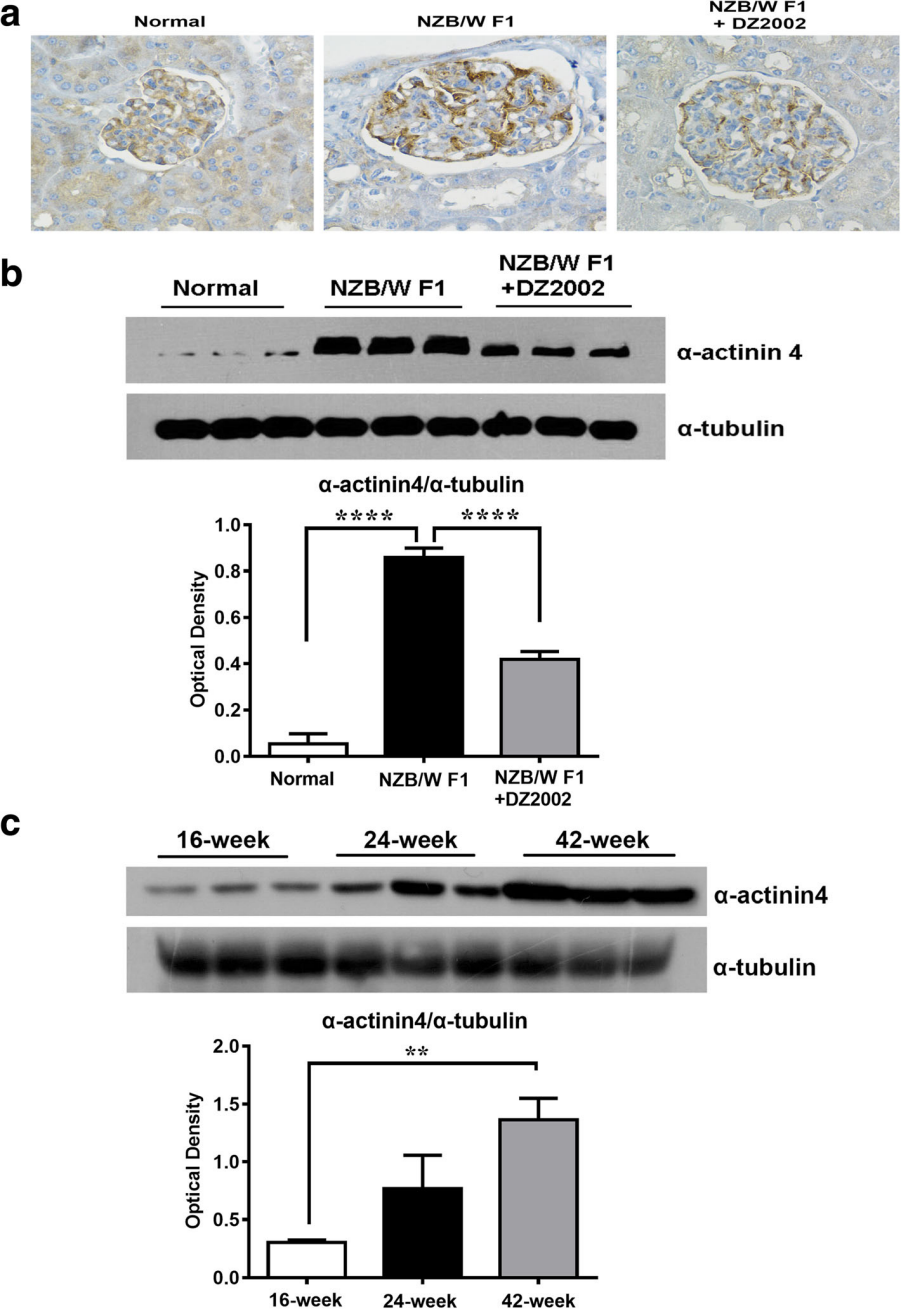
DZ2002 treatment reduced aberrant distribution and accumulation of α-actinin-4 in the glomeruli of NZB/WF1 mice. **a** Representative α-actinin-4 expression in glomeruli by microscopic viewing (× 400 magnification). Semi-quantitative measurement of α-actinin-4 in the kidney form mice of normal, NZB/WF1, and NZB/WF1+DZ2002 group (**b**) and from NZB/WF1 mice of indicated age (**c**). Protein level was measured from the kidney tissue lysate. Each blot represented data for individual mouse (*n* = 3 per group). Normal, age-matched female C57BL/6 mice; NZB/WF1, vehicle mice in the treatment experiment; and NZB/WF1+DZ2002, DZ2002-treated mice in the treatment experiment. Data were shown as the mean ± SD. **P < 0.01, ***P < 0.0001versus NZB/WF1 group or 16-week-old group

In light of a possible connection between α-actinin-4 and the rearrangement of the actin cytoskeleton in glomerulonephritis, we further examined the potential alteration in the abundance and distribution of α-actinin-4 in kidney. As shown in Fig. 6a, an aberrant distribution of α-actinin-4 was observed in the glomeruli of the NZB/W F1mice, compared with the normal mice. In normal glomeruli, α-actinin-4 staining revealed a moderate expression and uniform pattern, corresponding to its localization in the podocyte. However, lupus nephritis caused an altered distribution of α-actinin-4 from podocyte to the mesangial area with an aggregated pattern. DZ2002 treatment significantly decreased the accumulation of α-actinin-4 in glomeruli and rectified its disturbed localization. Quantitative determination exhibited that DZ2002 treatment reduced the expression of α-actinin-4 in the kidneys of lupus-prone mice (Fig. 6b). Moreover, we showed that the kidney abundance of α-actinin-4 dramatically increased in an age-dependent manner of NZB/W F1mice (Fig. 6c), suggested that aberrant α-actinin-4 accumulation could be a critical event in the progression of lupus nephritis.

### DZ2002 treatment restrained the elevated ILK expression and β1-integrin phosphorylation in the glomeruli of NZB/WF1 mice

**Fig. 7:**
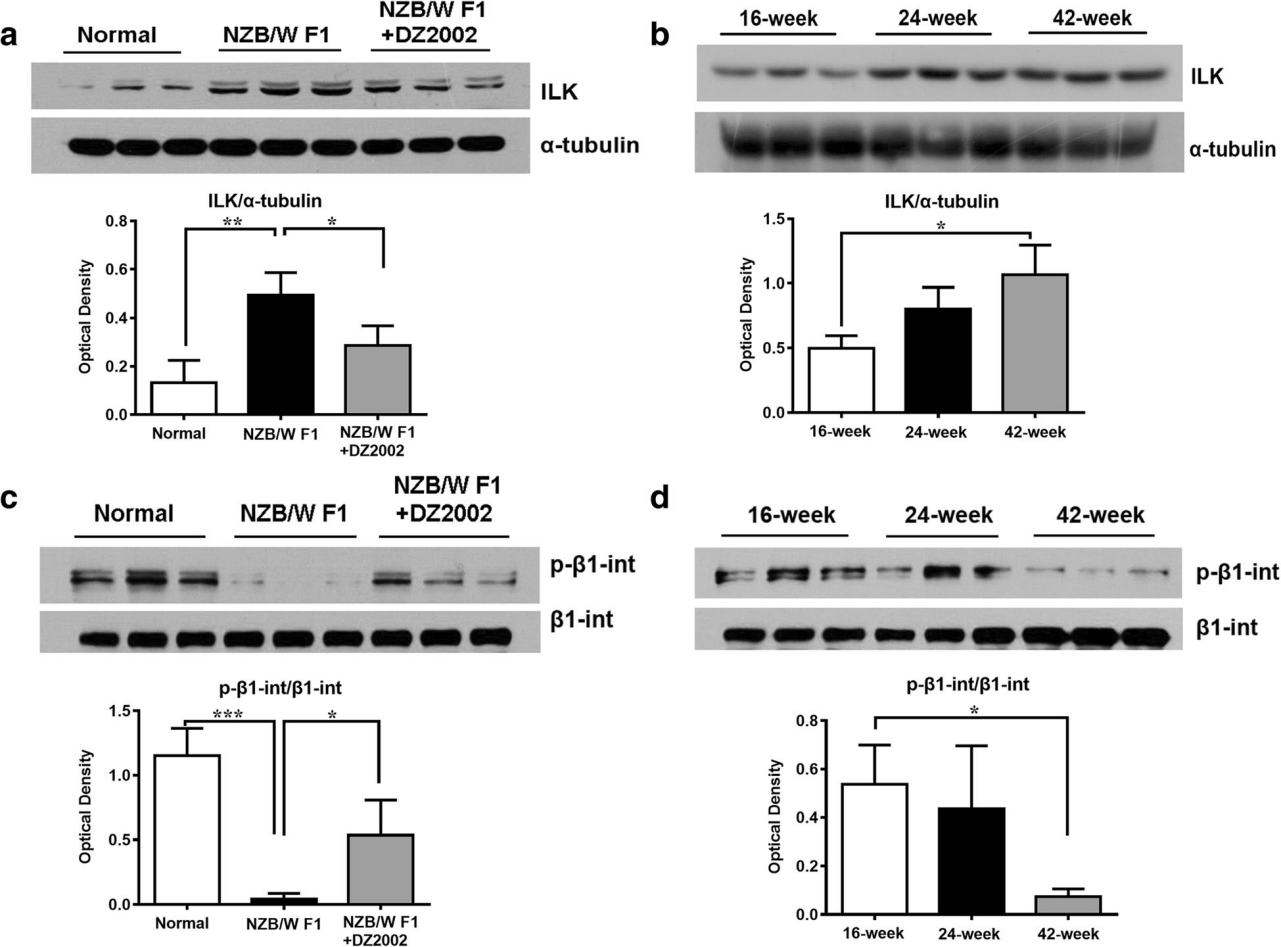
DZ2002 treatment restrained the elevated ILK expression and β1-integrin phosphorylation in the glomeruli of NZB/WF1 mice. Semi-quantitative measurement of ILK protein and phosphorylated β1-integrin in the kidney form mice of normal, NZB/WF1, and NZB/WF1+DZ2002 group (**a** and **c**) and from NZB/WF1 mice of indicated age (**b** and **d**). Protein level was measured from the kidney tissue lysate. Each blot represented data for individual mouse (*n* = 3 per group). Normal, age-matched female C57BL/6 mice; NZB/WF1, vehicle mice in the treatment experiment; and NZB/WF1+DZ2002, DZ2002-treated mice in the treatment experiment. Data were shown as the mean ± SD. *P < 0.05, **P < 0.01, and ***P < 0.001 versus NZB/WF1 group or 16-week-old group

The finding that the therapeutic benefits of DZ2002 was accompanied by decreased expression of α-actinin-4 in glomeruli prompted us to explore the impact of DZ2002 on the focal adhesion-associated proteins participates in cell-matrix interaction between podocyte and GBM. ILK is one of the essential components of cell-ECM adhesion and acts as a pseudokinase links integrins to the actin cytoskeleton and regulates focal adhesion assembly [39]. As shown in Fig. 7a, substantial increase in ILK protein level was observed in kidney of lupus-prone mice, compare with the normal ones, and DZ2002 administration significantly reversed the elevation of ILK. In addition, the ILK expression in kidney increased along with disease aggravation in NZB/WF1 mice (Fig. 7b). Similarly, induction of ILK has been previously demonstrated in podocyte insulted by various injurious stimuli, such as PAN, adriamycin (ADR), and high-ambient glucose condition [3].

Since ILK plays a key role in integrin-mediated cell adhesion and signaling, and ILK overexpression lead to reduced matrix adhesion, we next investigated the potential involvement of β1-integrin; the protein directly interact to ILK through its cytoplasmic domain. As shown in Fig. 7c, much lower phosphorylation state of β1-integrin appeared in the kidney of lupus-prone mice, compared with normal mice, and DZ2002 treatment significantly increased the phosphorylated form of β1-integrin. A gradual decrease of phosphorylated β1-integrin occurred in the kidneys of aged NZB/WF1 mice (Fig. 7d) and implied dysregulation of podocyte anchorage to the GBM in progressive glomerular failure.

### DZ2002 treatment restored podocyte number in the glomeruli of NZB/WF1 mice

**Fig. 8:**
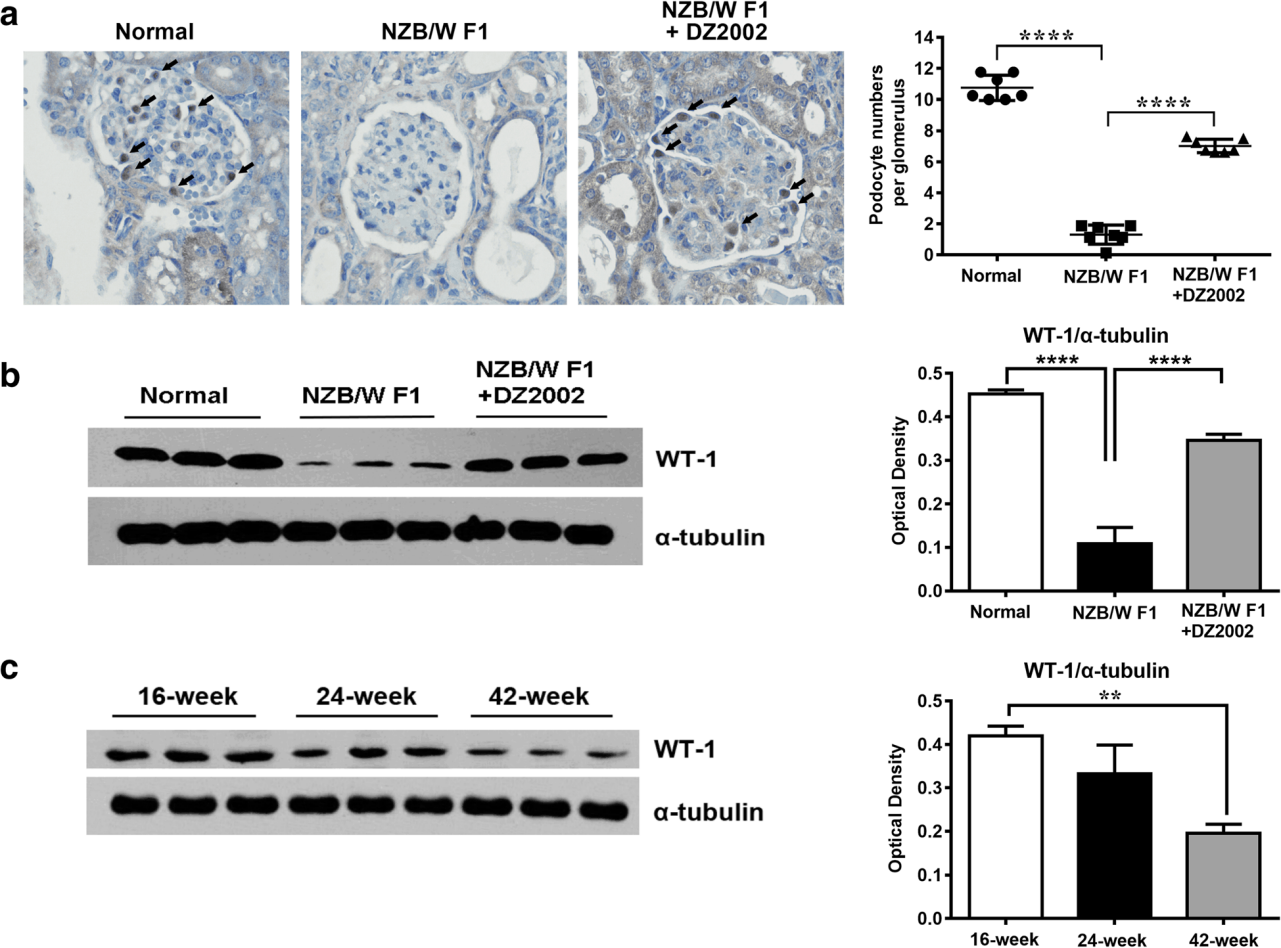
DZ2002 treatment restored podocyte number in the glomeruli of NZB/WF1 mice. **a** Left: Representative WT-1 expression in glomeruli by microscopic viewing (× 400 magnification). Right: Quantitative determination of the glomerular WT-1-positive podocyte numbers. Each dot represented data for individual mouse (*n* = 7 per group). Semi-quantitative measurement of WT-1 in the kidney form mice of normal, NZB/WF1, and NZB/WF1+DZ2002 group (**b**) and from NZB/WF1 mice of indicated age (disease stage) (**c**). Protein level was measured from the kidney tissue lysate. Each blot represented data for individual mouse (*n* = 3 per group). Normal, age-matched female C57BL/6 mice; NZB/WF1, vehicle mice in the treatment experiment; and NZB/WF1+DZ2002, DZ2002-treated mice in the treatment experiment. Data were shown as the mean ± SD. **P < 0.01, ****P < 0.0001 versus NZB/WF1 group or 16-week-old group

Because the α-actinin-4-associated cytoskeleton organization and ILK-regulated actin-integrin linkage are essential in mediating cell-matrix adhesion and cell survival [40, 41], we reasoned that the renal protective effects of DZ2002 attribute to the prevention of podocyte deletion and detachment from the GBM. To test this hypothesis, we examined the podocyte abundance in the kidneys of different groups. As shown in Fig. 8a and b, podocytes in the glomeruli were identified by the nuclear staining for transcription factor WT-1. Quantitative determination revealed that there was a marked reduction of WT-1-positive podocytes and WT-1 protein in the kidney of vehicle-treated NZB/W F1mice, and significantly recovered after DZ2002 treatment for 15 weeks. Western blot analysis demonstrated a disappearance of WT-1 protein in the kidneys of aged NZB/WF1 mice (42 weeks old), the ones suffered from severe glomerulonephritis as compared to the pre-diseased ones (16 weeks old) (Fig. 8c).

## Discussion

Nephritis is a frequent cause of SLE-associated morbidity and mortality. Renal involvement occurs in 40–70% of all patients with SLE [42]. Female NZB/WF1 mice are widely used as the model to investigate pathogenesis and potential therapeutic strategies for the human SLE. This strain of mice spontaneously develops an autoimmune syndrome with notable similarities to human SLE and invariably succumbs to severe glomerulonephritis by 12 months of age [43]. The renal lesion in lupus-prone mice progresses from an acute proliferative glomerulonephritis characterized by mesangial expansion and cellular infiltration to a chronic damage featured in glomerulosclerosis, interstitial fibrosis, and tubular atrophy, along with severe persistent proteinuria and fatal renal failure [44].

In the present study, we demonstrated that twice-daily administration of DZ2002 significantly alleviated the lupus nephritis and improved the renal function in NZB/WF1 mice, and the therapeutic benefits were accompanied by amelioration of proteinuria and glomerulosclerosis, as well as protection against podocytes depletion. Using LFQ proteomic strategy for the first time, we compared the murine kidney tissue proteomes between normal mice and lupus-prone mice treated with or without DZ2002, to investigate the mechanism of DZ2002 on autoimmune-mediated glomerulonephritis. A total of 3275 unique proteins were identified and quantified, in which 253 SCPs were screened for subsequent direct protein-protein interaction and GO analysis. KEGG pathway mapping revealed that key proteins in tight junction and focal adhesion processes varied between normal and lupus-prone mice, and significantly modulated by DZ2002 treatment in the diseased kidney tissues. Among the network of these two pathways, α-actinin-4, one of the SCPs in the kidneys from mice treated with or without DZ2002, functioned as central regulator of cytoskeleton and structural integrity of podocytes. Further Western blot and immunohistochemistry validations confirmed the pivotal events identified by the mass spectrometry-based LFQ analysis, including the downregulation of α-actinin-4 and ILK expression, as well as the upregulation of phosphorylation of β1-integrin in the kidney tissue after DZ2002 treatment.

Animal and human studies have demonstrated a strong correlation between defects in podocyte anchorage and several glomerular abnormalities. In podocyte, dynamic regulation of the actin cytoskeleton has been regarded as the center of focal adhesion-macromolecular cytoplasmic complexes anchor podocyte to GBM [45]. Previous studies showed alterations in α-actinin-4 levels in association with proteinuria in puromycin aminonucleoside (PAN)-induced nephrotic syndrome and in Masugi nephritis of rats [6, 8].

There is now increasing evidence that current therapies for nephrotic syndrome, including glucocorticoids and calcineurin inhibitor cyclosporin A (CsA), actually target the actin cytoskeleton in kidney podocytes, and their efficacy in nephritis might be independent of their immunosuppressive effects [46, 47]. Our results showed that DZ2002 treatment reduced aberrant distribution and accumulation of α-actinin-4 and restored the loss of podocytes in the glomeruli of NZB/WF1 mice. In line with our finding, results from other investigator demonstrated that mutant α-actinin-4 exhibited altered structural characteristics and localized abnormally; thus, interventions that modulate the expression or/and conformation of this cytoskeleton protein might be feasible to restore normal patterns of protein expression [48].

The interconnecting podocytes adhered to the GBM of the capillary wall function as a critical participant in formation of glomerular filtration barrier. The GBM is a dense network of secreted ECM components. Interactions between podocytes and the ECM are regulated by integrins. Upon integrin engagement of the ECM, there is integrin clustering and activation [49]. ILK acts as a central component of a heterotrimer (the PINCH-ILK-parvin complex) at ECM adhesions, and it interacts with the cytoplasmic domain of β1-integrin subunits and couples them to the actin cytoskeleton [39, 50]. The initial study reporting the discovery of ILK showed that it phosphorylates the cytoplasmic tail of β1 integrin. Recently, genetic analyses in mice revealed that the biological function of ILK is independent of its activity [51], and ILK is a bona fide pseudokinase according to the crystal structure results [52]. In present study, the elevated expression of ILK in the kidney of lupus-prone mice decreased significantly after DZ2002 treatment, in parallel to the alleviation of proteinuria and glomerulosclerosis.

Intriguingly, we observed a diminished phosphorylation pattern of β1-integrin in NZB/WF1 mice kidney along with the disease progression, and DZ2002 treatment restored the activation of β1-integrin in accordance with its anti-proteinuric effects. A functional investigation using directed mutagenesis in fibroblast cell line documented that the dephosphorylated form of β1-integrin promoted cell spreading, whereas a phosphorylated form of this protein promoted attachment to ECM [53]. This finding suggested that the therapeutic benefits of DZ2002 in lupus nephritis might be related to its ability to increase the anchoring strength of podocytes during injury.

Recent research of murine models and clinical studies provided evidence that the increased glomerular α-actinin expression after epithelial podocyte confluence play a critical role in renal pathology [54, 55], and emphasized the involvement of cross-reactivity of intraglomerular α-actinin and anti-dsDNA antibodies in glomerular immune complex formation [56, 57]. The alteration of α-actinin expression or structure that contributes to cellular transformation of renal parenchymal cell, including podocytes and mesangial cells, might increase the accessibility of both the membrane-associated and glomerular renal antigens to anti-dsDNA antibodies, especially for those specific to α-actinin. Given the post-translational modifications, including but not limited to acetylation, phosphorylation, and methylation, which regulate actin dynamics and are relevant to physiologic and pathologic processes [58], we speculated that DZ2002, the SAHH inhibitor, which could regulate macromolecular transmethylation, might interfere the methylation of particular sites of α-actinin molecules and contribute to the maintenance of structure stability. The precise mechanisms of how the effects elicited needed further exploration to elucidate the specific α-actinin residues susceptible to the inflammatory stressors and to the treatment of DZ2002.

In summary, our data indicated that alongside of attenuating glomerular immune complexes deposition and impeding pathologic lymphocytes polarization in SLE [18], the renal protective effects of DZ2002 may partly attributed to stabilization of the actin cytoskeleton and maintenance of podocyte numbers, in turn, are effective to improve proteinuria and kidney function in lupus nephritis.

## Conclusions

The present study demonstrated that the reversible SAHH inhibitor DZ2002 exerted its renal protective effects on lupus nephritis partly through stabilizing the focal adhesion of podocyte and GBM in glomeruli. Crosstalk between the podocyte actin cytoskeleton and focal adhesions is critical for maintaining the functions of glomerular filtration barrier. These findings might provide new insights into understanding that DZ2002, in addition to the immunosuppressive activities, could influence the structure and function of podocyte-GMB network in the kidney, suggested that directly modulation of podocyte biology could be a rational therapeutic intervention for glomerulonephritis in SLE. Further studies using super-resolution imaging and proteomic analyses of the adhesome in glomeruli are needed to fully characterize the molecular details on how the SAHH inhibitor influences the podocyte architecture and focal adhesion assembly.

## Additional files

Additional file 1: Supplementary Figures. (PDF 756 kb)

Additional file 2: Supplementary Tables. (XLSX 101 kb)

## Abbreviations

APC: Antigen-presenting cells
BUN: Blood urea nitrogen
ECM: Extracellular matrix
FASP: Filter-aided sample preparation
FDR: False discovery rate
FSGS: Focal segmental gomerulosclerosis
GBM: Glomerular basement membrane
GO: Gene ontology
HCA: Hierarchical clustering analysis
ILK: Integrin-linked kinase
LFQ: Label-free quantitative
LN: Lupus nephritis
MS: Mass spectrometry
NCE: Normalized collision energy
PAS: Periodic acid-Schiff
PPI: Protein-protein interaction
SAH: S-adenosyl-l-homocysteine
SAHH: S-adenosyl-l-homocysteine hydrolase
SAM: S-adenosylmethionine
SCP: Significantly changed protein
SDS: Sodium dodecyl sulfate
SLE: Systemic lupus erythematosus
TLR: Toll-like receptor
