# Supplementary material for: Reversible SAHH inhibitor protects against glomerulonephritis in lupus-prone mice by downregulating renal α-actinin-4 expression and stabilizing integrin-cytoskeleton linkage
Source: Arthritis Res Ther. 2019 Jan 29;21:40. doi: 10.1186/s13075-019-1820-3 (PMC6352376; doi:10.1186/s13075-019-1820-3)
Supplement: Supplementary file 1 — Supplementary Figures. (PDF 756 kb) [file 13075_2019_1820_MOESM1_ESM.pdf]

Title: Reversible SAHH inhibitor protects against glomerulonephritis in lupus-prone mice by regulating α-actinin-4 expression and integrin-cytoskeleton linkage

Authors:

Shijun He[†1,2], Xing Liu[†3], Zemin Lin[1], Yuting Liu[1,2], Lei Gu[3], Hu Zhou[*2,3], Wei Tang[*1,2], Jianping Zuo[*1,2]

Correspondence:

Hu Zhou, zhouhu@simm.ac.cn or Wei Tang, tangwei@simm.ac.cn or Jianping Zuo, jpzuo@simm.ac.cn

[†] Shijun He and Xing Liu contributed equally to this work.

[1]Laboratory of Immunopharmacology, State Key Laboratory of Drug Research, Shanghai Institute of Materia Medica, Chinese Academy of Sciences, Shanghai, 201203, China

[2] University of Chinese Academy of Sciences, No.19A Yuquan Road, Beijing 100049, China

[3]Department of Analytical Chemistry and CAS Key Laboratory of Receptor Research, Shanghai Institute of Materia Medica, Chinese Academy of Sciences, Shanghai, 201203, China

Supplementary Figure S1

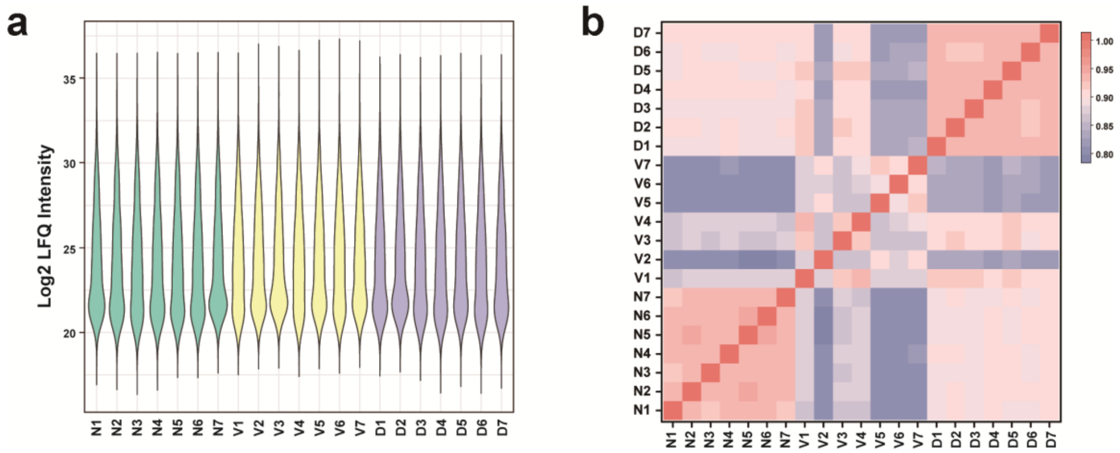

Fig. S1 Statistical analysis of the proteomics identified proteins. **a** The violin plot shows the LFQ intensity (log2) of all the identified proteins of three groups (n=7). **b** Correlation analysis all of samples. N: normal group, C57BL/6; V: model group, NZB/WF1; D: drug group, DZ2002 treated NZB/WF1**.**

Supplementary Figure S2

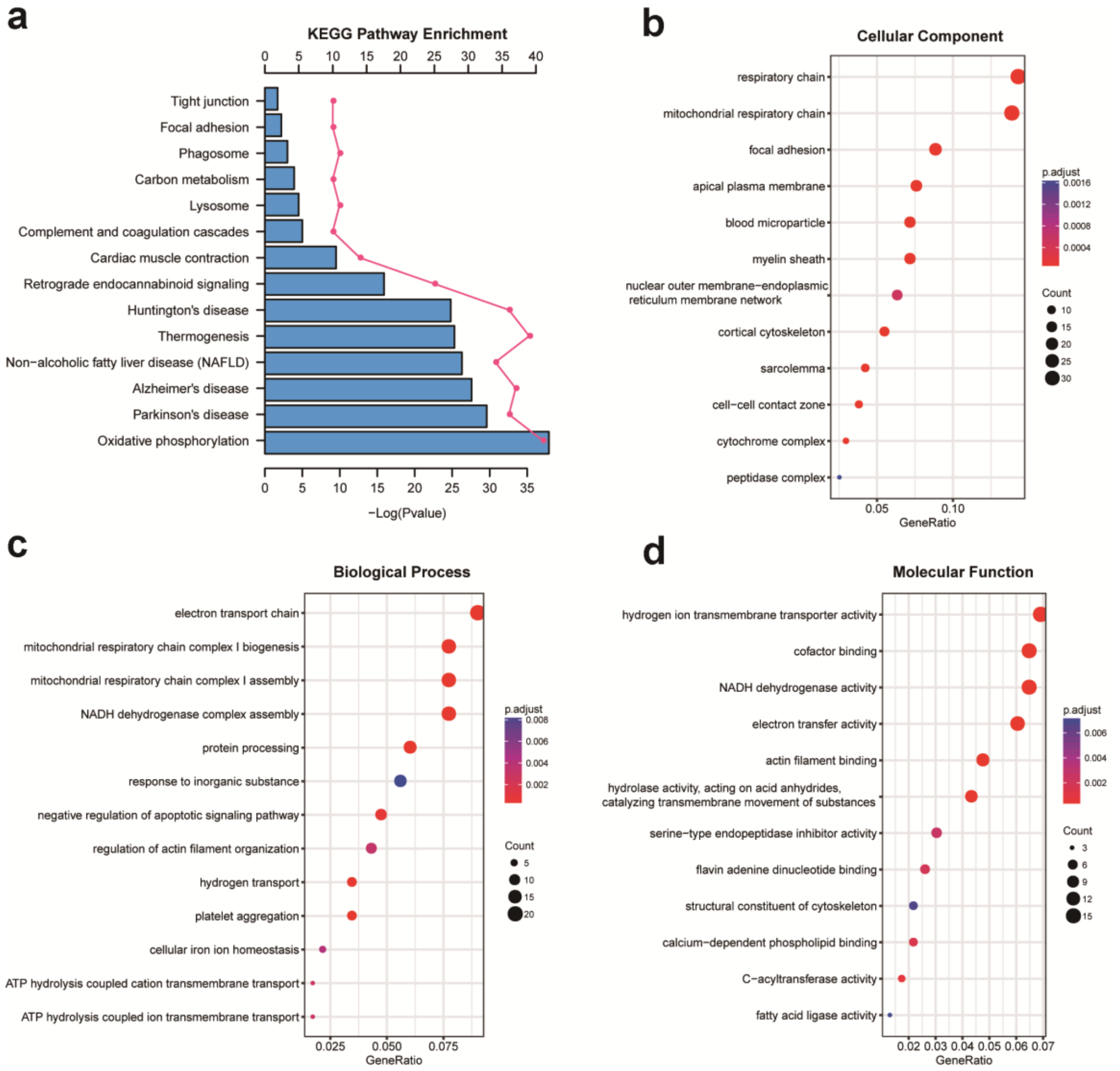

Fig. S2 Functional analysis of SCPs based on KEGG pathway and universal GO annotation terms. a Significantly changing proteins were mapped to the KEGG (Kyoto Encyclopedia of Genes and Genomes) pathway database (http://www.genome.jp/kegg/pathway.html). The enriched KEGG pathways were with p-value<0.05, and enriched SCPs numbers were more than ten. The bar plot shows the enriched pathways and the dot plot shows the enriched SCPs numbers.

b Gene ontology analysis of the percent of the identified proteins within cellular component annotations. **c** Same as (**b**) but for Gene ontology biological process annotations. **d** Same as (**b**) but for Gene ontology molecular function annotations.
